# The pelvic floor dysfunction: Where obstetrics meets urogynecology

**DOI:** 10.1111/aogs.70288

**Published:** 2026-06-12

**Authors:** Ingrid Volloyhaug, Pia Teleman, Dudley Robinson

**Affiliations:** ^1^ Department of Clinical and Molecular Medicine Norwegian University of Science and Technology Trondheim Norway; ^2^ Department of Obstetrics and Gynecology Trondheim University Hospital Trondheim Norway; ^3^ Department of Women's and Children's Health Karolinska Institutet Stockholm Sweden; ^4^ Department of Women's Health and Health Professions Karolinska University Hospital Stockholm Sweden; ^5^ King's College Hospital London UK

Pelvic floor dysfunction, as an umbrella term for urinary incontinence, anal incontinence, pelvic organ prolapse, pelvic pain or sexual dysfunction, commonly begins following childbirth. Vaginal birth, multiparity and significant perineal tears are known risk factors for long‐term symptoms. Trauma to the levator ani muscles, anal sphincters, nerves, connective tissue fascia and ligaments might contribute to development of pelvic floor disorders in the short and long term after delivery. Other conditions that increase the strain on the pelvic floor, that is, obesity, constipation, chronic cough and strenuous physical activity may also have a compound effect on childbirth related injury.

Pelvic floor dysfunction often results in both physical limitations and a reduced sense of control. In this issue, Milsom and coauthors in their state‐of‐the‐art review conclude that pelvic floor disorders incur a huge socioeconomic burden on women throughout the world and that management strategies during delivery should be individualized to reduce the burden of pelvic floor dysfunction following vaginal birth.

Increased awareness and knowledge about how to prevent trauma during childbirth is essential to reduce the risk of future pelvic floor disorders. New diagnostic tools that help the diagnosis and understanding of mechanisms that cause dysfunction are required, and this will also improve treatment strategies for symptomatic women.

In this issue of the AOGS, we have invited researchers with a special interest in different aspects of the pelvic floor in relation to childbirth and urogynecology to publish their results. The papers mainly focus on three areas: *Perineal trauma* and associated symptoms in the short and long term, *new ultrasound techniques* of the levator ani and anal sphincters during childbirth and postpartum as well as *surgical procedures* for treatment of urinary incontinence and pelvic organ prolapse.

## 1 PERINEAL TRAUMA

Perineal tears, especially obstetric anal sphincter injury (OASI), have been studied regarding healing, imaging, and outcome in the short‐term, but also in the longer term, showing a significantly increased risk of anal incontinence.^1^ During the first year postpartum third‐ and fourth‐degree tears result in increased pain and delay of resuming sexual activity for a longer time compared to minor tears.^2^

Recently, research has also focused on second‐degree tears. Several studies have highlighted the need for subclassification and more detailed description of the perineal structures involved in the tear and to what extent. Research regarding the outcome of second‐degree tears is challenged by the lack of reliable data from larger populations.

Studies on early complications such as infection with or without wound dehiscence and the best way to treat them are important. Does it matter whether we resuture a dehiscence or leave it to secondary healing? If so, how do we organize follow‐up after perineal tears? Episiotomies have been demonstrated to inflict more pain than second‐degree tears during the first months postpartum and also more infections. Therefore, more research is needed, especially in relation to the vast variation in practice and in relation to protection against OASI in primipara.^3^ We also need to study the optimization of healing and enhancing pelvic floor function after delivery.

It is our view that much more can be done in preventing, diagnosing, suturing, and rehabilitating perineal injuries in order to enhance women's pelvic floor health throughout life.

## 2 IMAGING

*Transperineal ultrasound* has emerged as an important tool for examination of the levator ani complex and the anal sphincters over the past 15 years. This has also given new insight about the robust association between levator trauma and pelvic organ prolapse and has established forceps as an important risk factor for such injuries.^4^ Pelvic floor muscle exercises are used in treating pelvic floor disorders. Traditional methods for assessment of contraction, such as digital palpation and perineometry, are biased by the training of the examiner and coactivation of other muscles. Luo and coauthors found that *transperineal ultrasound* demonstrated high reliability and provided an objective, non‐invasive method for assessing pelvic floor muscle contractility, confirming the results from previous similar studies. *Transperineal ultrasound* was also used by Lees and coauthors to highlight the understanding of pelvic floor dimensions in the passive second stage of labor and in studying the relationship between the levator muscle and maternal age, BMI, ethnicity, and gestational age.

Using a high‐frequency *transvaginal probe*, Youssef and coauthors found feasible and highly reproducible measurement of the anterior external anal sphincter. This could be an easier and more available method for gynecologists to assess the anal sphincters and needs further evaluation. *Endoanal ultrasound* has traditionally been less available for gynecologists as a diagnostic tool. In this issue, Rotstein and coauthors use three‐dimensional endoanal ultrasound measurements for the assessment of perineal body size in a cohort of Swedish nulligravidae, and they establish a normal range that can be used as reference in future research.

## 3 TREATMENT STRATEGIES

The role of vaginal estrogen therapy in the management of pelvic floor dysfunction following the menopause is well established although far less is known about the effectiveness in women who are breast feeding, and therefore hypo‐estrogenic, in the post‐natal period. The utility of postpartum vaginal estrogen treatment is addressed in this special issue although the effect of adjuvant vaginal estrogen therapy on surgical outcomes remains unclear.^5^

While all women will initially benefit from a conservative approach to pelvic floor dysfunction in the postpartum period many will ultimately benefit from surgical intervention for stress urinary incontinence or urogenital prolapse. Over the last ten years, the role of the use of mid‐urethral tapes and vaginal mesh for the management of stress urinary incontinence and pelvic organ prolapse has become increasingly controversial with this surgery currently “paused” in some countries and banned in others. Consequently, there has been renewed interest in traditional retropubic procedures such as colposuspension as well as an acknowledgment of the longer‐term morbidity associated with mid‐urethral sling procedures and vaginal mesh procedures. This is also reflected in a shift to traditional native tissue surgery and well‐established uterine sparing techniques such as the Manchester Repair.

Perhaps equally controversial is the use of urodynamic investigations prior to continence surgery^6^ and the use and rationalization of pre‐operative investigations is covered in this issue by a survey of the Nordic countries.

And finally, Hubner and coauthors have conducted a state‐of‐the‐art review article summarizing important future directions regarding pelvic floor in obstetrics and urogynecology. Four major work packages have been identified. 1. Epidemiology and risk stratification. 2. Mechanistic and translational research. 3. Diagnostic and preventive strategies. 4. Implementation and knowledge translation.

Pelvic floor dysfunction remains an important and often underrecognized, and poorly managed, sequalae of childbirth. A better understanding of etiology in addition to multidisciplinary cooperation between obstetricians, urogynecologists, colorectal surgeons, and allied health professionals should lead to improved outcomes for all of our new mothers who experience pelvic floor trauma at the time of delivery. In addition, greater recognition and effective management should prevent the long‐term sequalae of this often‐devastating condition.
